# Case report: *KPTN* gene-related syndrome associated with a spectrum of neurodevelopmental anomalies including severe epilepsy

**DOI:** 10.3389/fneur.2022.1113811

**Published:** 2023-01-10

**Authors:** Svea Horn, Magdalena Danyel, Nina Erdmann, Felix Boschann, Cecilia Gunnarsson, Saskia Biskup, Jerome Juengling, Cornelia Potratz, Christine Prager, Angela M. Kaindl

**Affiliations:** ^1^Department of Pediatric Neurology, Charité–Universitätsmedizin Berlin, Berlin, Germany; ^2^Center for Chronically Sick Children, Charité–Universitätsmedizin Berlin, Berlin, Germany; ^3^Institute of Medical Genetics and Human Genetics, Charité–Universitätsmedizin Berlin, Berlin, Germany; ^4^BIH Biomedical Innovation Academy, BIH Charité Clinician Scientist Program, Berlin Institute of Health at Charité–Universitätsmedizin Berlin, Berlin, Germany; ^5^Department of Clinical Genetics, Linköping University, Linköping, Sweden; ^6^Department of Biomedical and Clinical Sciences, Linköping University, Linköping, Sweden; ^7^Centre for Rare Diseases in South East Region of Sweden, Linköping University, Linköping, Sweden; ^8^Praxis für Humangenetik Tübingen, Tübingen, Germany; ^9^Institute for Cell Biology and Neurobiology, Charité–Universitätsmedizin Berlin, Berlin, Germany

**Keywords:** *KPTN* gene, macrocephaly, neurodevelopment delay, epilepsy, splice site variant

## Abstract

Biallelic variants in the kaptin gene *KPTN* were identified recently in individuals with a novel syndrome referred to as autosomal recessive intellectual developmental disorder 41 (MRT41). MRT41 is characterized by developmental delay, predominantly in language development, behavioral abnormalities, and epilepsy. Only about 15 affected individuals have been described in the literature, all with primary or secondary macrocephaly. Using exome sequencing, we identified three different biallelic variants in *KPTN* in five affected individuals from three unrelated families. In total, two *KPTN* variants were already reported as a loss of function variants. A novel splice site variant in *KPTN* was detected in two unrelated families of this study. The core phenotype with neurodevelopment delay was present in all patients. However, macrocephaly was not present in at least one patient. In total, two patients exhibited developmental and epileptic encephalopathies with generalized tonic-clonic seizures that were drug-resistant in one of them. Thus, we further delineate the *KPTN*-related syndrome, especially emphasizing the severity of epilepsy phenotypes and difficulties in treatment in patients of our cohort.

## 1. Introduction

Biallelic variants in *KPTN* cause the syndrome referred to as autosomal recessive intellectual developmental disorder 41 (MRT41, OMIM 615637) (1). MRT41 is characterized by developmental delay, predominantly in language development, behavioral abnormalities, and epilepsy. However, only few affected individuals have been described so far. MRT41 was first identified in four consanguineous families of the Amish community of Ohio in 2014 (1). All affected individuals in this study presented with macrocephaly and neurodevelopmental delay, and one-third developed epilepsy with the absence of seizures and generalized tonic-clonic seizures. Using microsatellite–marker analysis followed by whole exome sequencing (WES), the homozygous truncating variant p.(Ser259^*^) in the *KPTN* gene (NM_007059.4) was discovered in two families of this study (1). In the two other families of this study, the above-mentioned nonsense variant was detected in a compound heterozygous state with an in-frame duplication p.(Met241_246Glndup). This duplication of the six amino acids was predicted to disrupt the alpha-helix 4 of the protein. Functional studies of both variants suggested a loss of function effect (LoF) either by the degradation of the mutant transcript *via* nonsense-mediated mRNA decay (NMD) or by the production of mislocalized and/or non-functional protein products (1). This duplication together with the splice site variant c.394+1G>A, and a frameshift variant (2) p.(Ser223fs), was described in two further affected families, respectively (2, 3). An additional recurrent homozygous frameshift variant p.(Ser200Ilefs^*^) was reported in two unrelated families (4, 5). All these affected individuals presented with core manifestations, developmental delay, and macrocephaly (4, 5).

The *KPTN* gene encodes for kaptin, which plays a crucial role in the neuromorphogenesis and development of the brain. Kaptin was shown to be expressed in neuronal cells and was found to localize to F-actin-rich structures (1). Immunofluorescence analyses in primary neuronal cells demonstrated endogenous and GFP-tagged kaptin to be associated with dynamic actin cytoskeletal structures (1). The loss of kaptin function leads to an impairment of the neuronal actin cytoskeleton, dendritic arborization, and spine formation (1). Furthermore, kaptin has been proven as a negative upstream regulator of the mTORC1 signaling pathway as a part of the KICSTOR protein complex (6). mTORC1 is an important regulator of cell growth and is dysregulated in different human diseases, including cancer and epilepsy phenotypes. Because of hyperactive mTORC1 signaling, patients with pathogenic variants in *KPTN* might benefit from mTORC inhibitors (5).

Detailed data on seizures and antiepileptic treatment of individuals with biallelic pathogenic variants in *KPTN* have not yet been published. We present the data of 5 novel patients with special emphasis on severe epilepsy and antiepileptic treatment in two of them.

### 1.1. Clinical report

**Figure 1:**
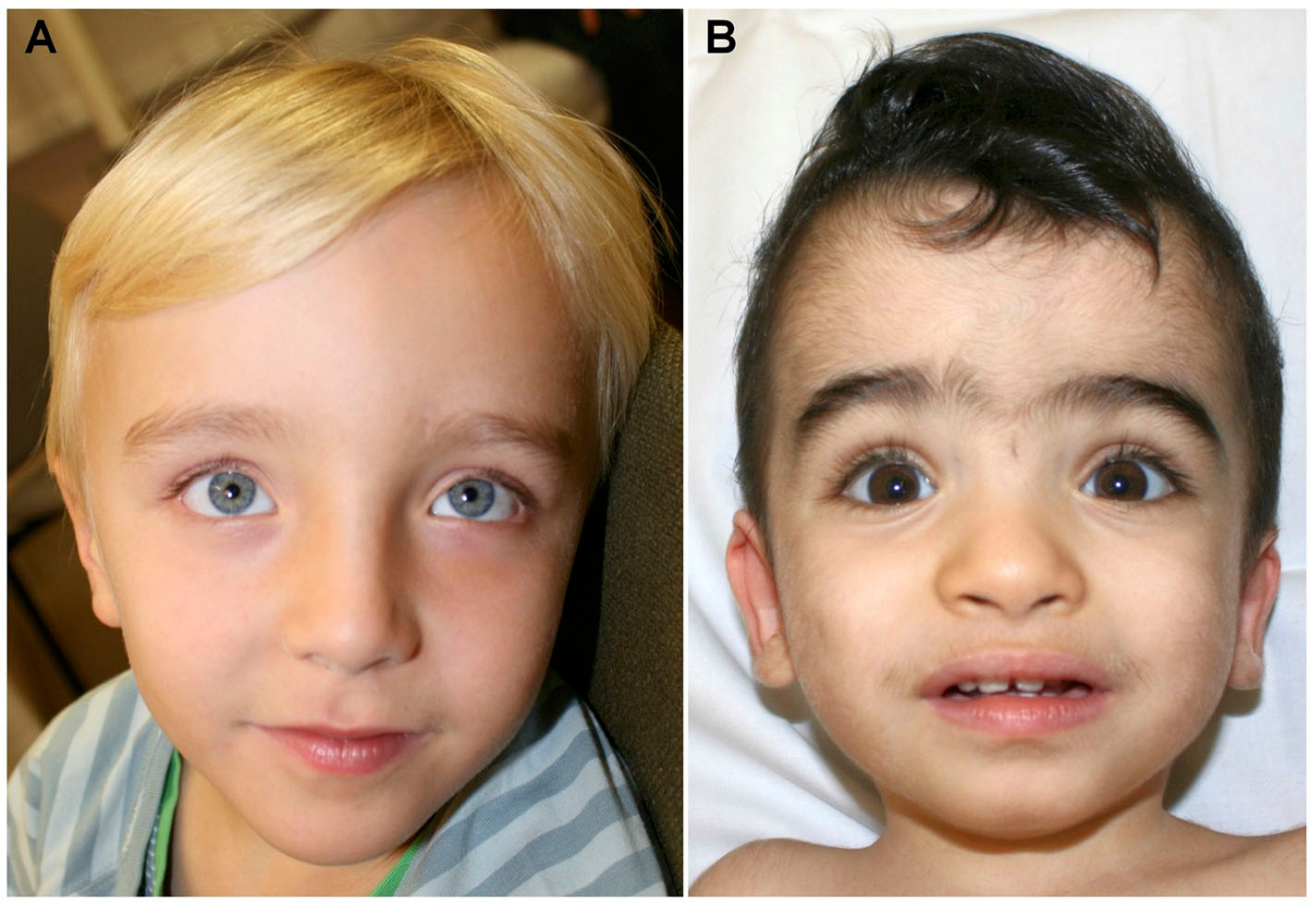
**(A)** Facial aspect of patient 1 at the age of 6 years showing prominent forehead and hypertelorism. **(B)** Face of patient 2 at the age of 18 months. Prominent forehead, synophris, hypertelorism and mildly downward slanting palpebral fissures.

**Patient 1** was a 6-year-old son of non-consanguineous parents of German origin. He was born at term without complications after an uneventful pregnancy, with normal birth weight (2,710 g, −0.9 SD), birth length (49.0 cm, −0.5 SD), and occipitofrontal head circumference (OFC, 34.5 cm, +0.1 SD). Secondary disproportionate macrocephaly and facial dysmorphisms including a prominent forehead, frontal bossing, and thin lips were identified at 2 months of age (Figure 1A, Table 1). The OFC was +2.2 SD (55 cm) at 6 years of age, while height and weight were both at −1 SD. Motor development was normal with rolling from side to side at the age of 9 months and free ambulation at 18 months of age. Cognitive and speech development were delayed already early. During the 1st year, syllables were uttered, and a few single words were spoken inconsistently by the first birthday. Speech comprehension was better than expressive speech. At the age of 6 years, the boy spoke about 50 words inconsistently in one to two-word sentences. At this age, the patient presented with autistic features such as repetitive movements, poor eye contact, and restlessness. Standardized cognitive testing was not feasible due to severe intellectual impairment. The patient received speech and occupational therapy.

**Table 1:** Comparison of clinical manifestations of *KPTN* gene-related syndrome in five novel patients with previously reported patients.

**Patients**	**Patient 1**	**Patient 2**	**Patient 3**	**Patient 4**	**Patient 5**	**Data in literature^a^** ***n* = 15**
Origin	Germany	Afghani	Syria	Syria	Syria	
Sex	Male	Male	Female	Male	Male	8 male/7 female
Age at evaluation	6 years	16 months	13 years	9 years	6 years	
Genotype in *KPTN*	c.597_598dupTA; p.Ser200Ilefs^*^55 c.714_731dup; p.Met241_Gln246dup	homozygous c.599+1G>A	homozygous c.599+1G>A	homozygous c.599+1G>A	homozygous c.599+1G>A	
ACMG classification	Both pathogenic	Pathogenic	Pathogenic	Pathogenic	Pathogenic	
**Anthropometric data**						
Head circumference (SD) at birth	34.5 cm (+0.1 SD) (40th week)	31 cm (-0.2 SD) (33rd week)	NA	NA	NA	Macrocephaly 45%
Head circumference at last evaluation (SD)	55 cm (+2.2 SD)	48 cm (+0.02 SD)	NA	NA	NA	Macrocephaly 100%
Weight at last evaluation (SD)	19 kg (−1 SD)	8.7 kg (−1.7 SD)	40 kg (−1.1 SD)	30 kg (−0.1 SD)	NA	NA
Height at last evaluation (SD)	112.5 cm (−1.2 SD)	78 cm (−0.8 SD)	150 cm (−1.3 SD)	128 cm (1.4 SD)	NA	NA
Dysmorphic facial features	Frontal bossing	Frontal bossing	Prominent chin	-	NA	Frontal bossing 100%, prominent chin 73%
**Neurodevelopmental phenotype and epilepsy**						
Developmental delay/Intellectual disability	+	+	+ (IQ 30–50)	+ I(IQ 50–70)	+ (IQ 50–70)	100%
Behavioral abnormalities	Repetitive movements, autistic features, stereotypic movements	Stereotypic movements	Behavioral abnormalities	ND	ND	33%
Epilepsy	+ (GTCS)	+ (GTCS)	–	–	–	33% (GTCS *n* = 3, AS/GTCS *n* = 2),
Onset of seizures in years	5 years	13 months	–	–	–	3 months−7 years
EEG findings	Epileptic discharges, focal slow activity frontal	Front-otemporal sharp-wave complexes on the left side	ND	ND	ND	NA
MRI findings	Optical nerve tortuosity, flat pituitary gland	Mild enlargement of the inner cerebrospinal fluid spaces	Normal	ND	ND	Different brain abnormalities 50%

ND, not documented; NA, not available; CTCSs, generalized tonic-clonic seizures; Ass, absence seizures;

a (1–5).

**Figure 2:**
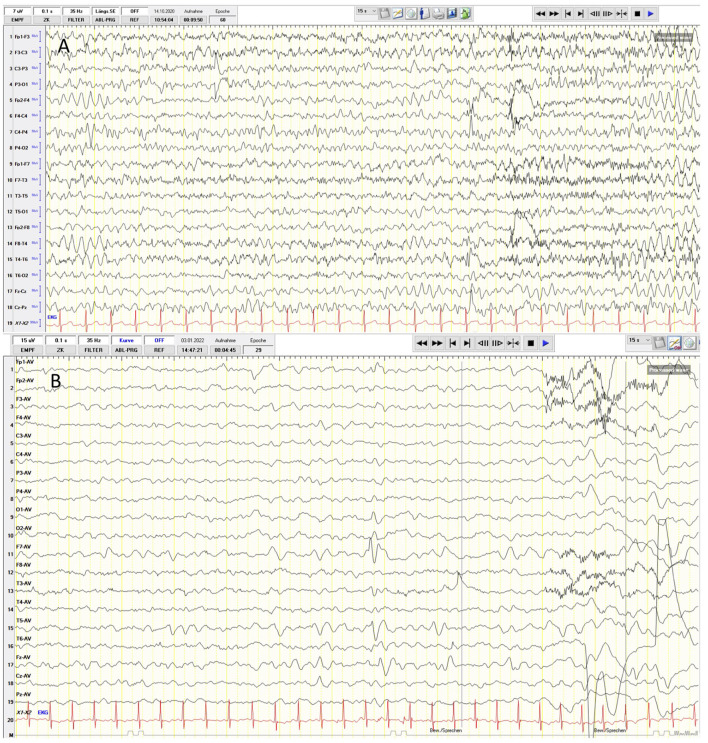
**(A)** Routine electrophalography (EEG) reveals focal sharp-waves (left, frontoparietal), rhythmic 6 Hz electrical activity and signs of focal cerebral dsfunction with a frontal theta-waves slow activity in the frontal area. **(B)** The EEG examination of patient 2. Frontotemporal sharp-wave complexes on the left side and rhythmical 4–5 Hz activity.

Generalized tonic-clonic seizures (GTCSs) without awareness were observed predominantly at night starting at the age of 5 years. In the beginning, the GTCS lasted for 30 s and were accompanied by upward gaze and postictal tiredness. Routine electroencephalography (EEG) revealed the focal sharp waves (left, front-oparietal), rhythmic 6 Hz electrical activity, and signs of focal cerebral dysfunction with theta waves slow activity in the frontal area (Figure 2A). Antiseizure medication (ASM) with levetiracetam (40 mg/kg/day) was implemented. Nevertheless, nightly GTCSs were observed in series (each 30 s, with regaining awareness in between) and episodes during sleeping with lip-smacking. Therefore, the dosage of levetiracetam was increased to 60 mg/kg/day and clobazam was added with 0.11 mg/kg/day. Shortly thereafter, painful muscle cramps in the feet occurred but were not associated with any epileptic discharges on EEG. Given that muscle cramps can occur as a side effect of clobazam, clobazam was weaned and oxcarbazepine was introduced (0.25 mg/kg/day). Unfortunately, this change in ASM led to an increase in short episodes of lip-smacking, teeth grinding, upward gaze, dizziness, apathy, and fatigue. After weaning of oxcarbazepine, the patient had to be hospitalized four times because of recurrent status epilepticus (SE), which presented as a series of focal onset seizures (stiffness of arm or leg without losing awareness) and partially secondary GTCS with oxygen desaturation to 75% and perioral cyanosis. The patient was admitted one time to the intensive care unit (ICU) and treated acutely with oral midazolam (5 mg), intravenous (IV) lorazepam (0.1 mg/kg), IV lacosamide (2 mg/kg), IV levetiracetam (60 mg/kg), IV clonazepam (0.06 mg/kg), and finally continuous IV fosphenytoin (exact dosage not available) without success. Therefore, continuous IV phenobarbital (10 mg/kg/day) was introduced after 24 h of SE, and the seizures were stopped. At a phenobarbital dosage of IV 10 mg/kg/day, bradypnea with desaturations occurred. Phenobarbital was, thus, given orally (5 mg/kg/day), but had to be weaned since the patient developed a severe skin rash and elevated liver enzymes [GPT 670 U/I (normal range < 41 U/I) and GOT 280 U/I (normal range < 50 U/I)]. Instead, oral valproic acid was implemented (10 mg/kg/day). Finally, consultation at the age of 6.5 years, the patient had been seizure free for 6 months on the combination of four ASMs (levetiracetam 60 mg/kg/day, clonazepam 0.06 mg/kg/day, valproic acid 10 mg/kg/day, and lacosamide 2 mg/kg/day). The course of the disease leads to the classification of developmental and epileptic encephalopathies.

**Figure 3:**
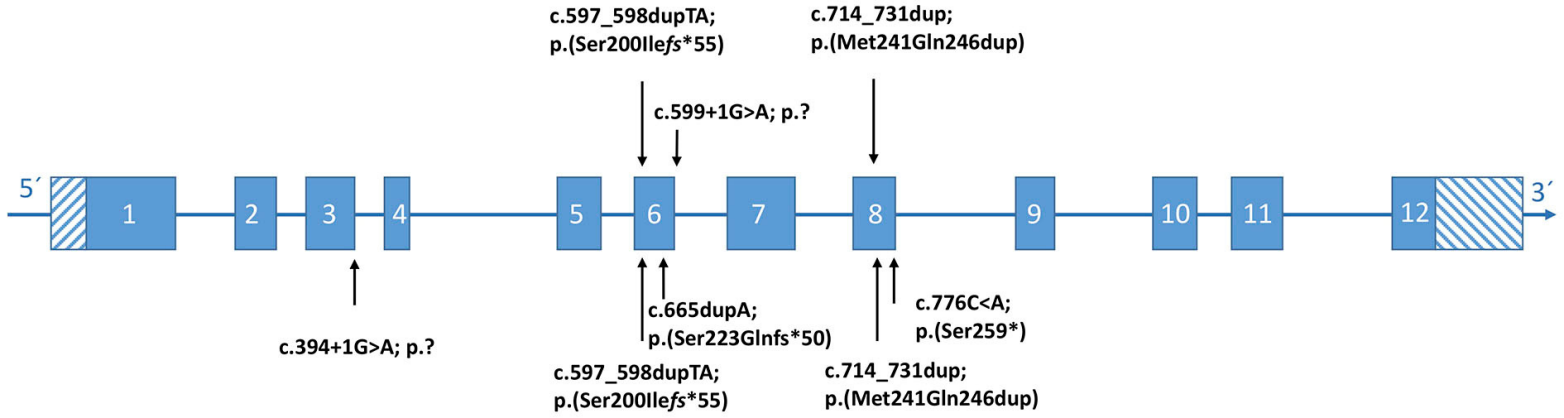
Position of KPTN (NM007059.4) variants identified in this study **(top)**, with respect to the affected exon. **(Bottom)** Reported pathogenic variants according to the literature.

Cranial magnetic resonance imaging (MRI) revealed the optical nerve tortuosity and a flat pituitary gland, which could be interpreted as indirect signs of benign intracranial hypertension. In the lumbar puncture, the opening pressure was borderline with 24 cmH20 (7, 8), and there were no abnormalities on the ophthalmological examination. The patient did not present with the clinical signs of intracranial hypertension such as vomiting or headaches. The results of chromosome analysis and array comparative genetic hybridization (CGH) were normal. WES detected compound heterozygous pathogenic variants in the *KPTN* gene (c.597_598dupTA https://www.ncbi.nlm.nih.gov/clinvar/variation/279826; p.Ser200Ilefs^*^55 and c.714_731dup https://www.ncbi.nlm.nih.gov/clinvar/variation/279826; and p.Met241_Gln246dup) (Figure 3). The American College of Medical Genetics and Genomics (ACMG) classification of both variants is found in the Supplementary material.

They co-segregated with the phenotype in the family.

**Patient 2** was a 1.5-year-old male child of consanguineous, healthy parents of Afghani origin. A maternal uncle had died 2 days postnatally of an unknown cause and a paternal uncle had febrile seizures. The pregnancy was complicated by pre-eclampsia and gestosis resulting in premature birth by Cesarean section at 33 + 1 weeks of gestation. His anthropometric data were still normal at birth, with a weight of 1,605 g (−1.2 SD), a length of 43 cm (−0.6 SD), and an OFC of 31 cm (−0.2 SD). Facial dysmorphic features comprised of the prominent forehead, synophrys, a low frontal hairline, and dysplastic ears but no macrocephaly (Figure 1B, Table 1). The boy had an accessory mamilla and seven café-au-lait-spots. The patient was treated in the neonatology ICU until the 18th day of life due to prematurity. Here, an atrial septal defect II and patent foramen ovale were diagnosed, but no complications occurred. Cardiologic examinations were carried out on a regular basis. His motor development was delayed. He began to crawl at the age of 13 months but was unable to walk without support at the last consultation at the age of 16 months. At that time, he spoke two words. His socioemotional development and play behavior were not age-appropriate, and stereotypical movements of hands, especially “hand-wringing movements” were observed. At the age of 16 months, his height was 78 cm (−0.8 SD), weight was 8.7 kg (−1.7 SD), and OFC was 48 cm (+0.02 SD).

At the age of 13 months, the patient was hospitalized for GTCS without awareness, upward gaze, and with oxygen desaturation as low as 57% as well as perioral cyanosis. The EEG examination was pathological due to frontotemporal sharp-wave complexes on the left hemisphere and a rhythmic 4–5 Hz activity (Figure 2B). ASM with levetiracetam (40 mg/kg/day) was initiated but due to aggressive behavior, it had to be replaced with lamotrigine (5.5 mg/kg/day). No seizures occurred after treatment initiation.

Brain MRI showed no anomalies other than a marginal enlargement of the inner cerebrospinal fluid spaces. This was in line with an elevated cerebrospinal fluid opening pressure of 50 cm H20 in a lumbar puncture. No signs of papilledema were found in the ophthalmological examination. Treatment with acetazolamide (10 mg/kg/day) was implemented, and in the further course, the lumbar puncture opening pressure decreased to 19 cm H20.

Chromosome analysis and array CGH revealed normal results. Trio-WES was performed and detected the homozygous splice site variant *KPTN* (c.599+1G>A) https://www.ncbi.nlm.nih.gov/clinvar/variation/1706595. The c.599+1G>A variant is predicted to result in aberrant splicing (Figure 3). The ACMG classification is found in the Supplementary material.

The mutation was co-segregated in the respective family. With respect to the high number of café-au-lait-spots, an additional targeted analysis of the *NF1* and *SPRED1* genes was performed and revealed negative results.

**Patient 3** was a 13-year-old girl, the first child of consanguineous parents who were first cousins from Syria. Her psychomotor development was markedly delayed from infancy (Table 1). At the age of 13 years, she had a severe intellectual disability with an IQ of 30–50 without speech development. She had behavior problems such as autistic features. In addition, she showed profound sensorineural hearing loss. At the age of 13 years, her height was 150 cm (-1.3 SD), and her weight was 40 kg (-1.1 SD). Her facial signs included a broad nasal bridge, a short philtrum, a large mouth, and a prominent chin. MRI of the brain showed no abnormalities. WES revealed the homozygous variant c.599+1G>Ain *KPTN* (Figure 3). In addition, the homozygous pathogenic variant c.35delG in *GJB2*, associated with hearing loss, was identified. Neither MRI nor EEG results have been available for evaluation.

**Patients 4 and 5** were her affected brothers. Both were born at 40 weeks of gestation with normal measurements and have no medical problems in the neonatal period. Patient 4 was a 9-year-old boy with psychomotor delay and moderate intellectual disability with an IQ of 50–70 (Table 1). The expressive language was particularly affected. Physical examination at the age of 9 years showed a height of 128 cm (−1.4 SD), a weight of 30 kg (−0.1 SD), and no facial abnormalities. Hearing tests gave normal results. Patient 5 was a 6-year-old boy who also showed moderate intellectual disability, especially with difficulties in expressive language. A hearing problem was excluded in this patient. A genetic investigation confirmed the presence of the homozygous variant c.599+1G>A in *KPTN* in both brothers (Figure 3). Neither of them had seizures. The parents were both heterozygous carriers of the pathogenic *KPTN* c.599+1G>A variant and the *GJB2* c.35delG variant, respectively.

## 2. Methods

The institutional ethics committee of the Charité did not require the study to be reviewed or approved by an ethics committee because all investigations were standard di for WES in patients 1 and 3–5, sequencing libraries were prepared for each sample from 50 ng genomic DNA using the Twist enrichment workflow (Twist Bioscience, San Francisco, CA) and a custom-design enrichment probe set (CeGaT ExomeXtra 1.0) or SureSelectXT workflow (Agilent, Santa Clara, CA) and the Human All Exon enrichment kit (version 6), respectively. Library preparation and capture were performed according to the manufacturer's instructions, and paired-end sequencing was performed on a NovaSeq instrument (Illumina, San Diego, CA) with 2 × 100 base pairs (bp) read length. After demultiplexing (Illumina bcl2fastq), adapters were trimmed with Skewer. Trimmed raw reads were aligned to the human genome (hg19) with the Burrows–Wheeler Aligner. Average coverage on target was 155× and 158×, respectively. Sequence variants were called (VarScan 2.4.2, CeGaT extended version or CeGaT stratacall, respectively) with a minimum variant allele frequency of 5%. Resulting variants were annotated with population frequencies from dbSNP, gnomAD (2.1/3.1), or ExAC (0.3.1), respectively, and an internal database (CeGaT) with functional predictions from dbNSFP, with the publications from HGMD available at the time, and with transcript information from Ensembl, RefSeq, Gencode, and CCDS. Variants were filtered to remove the frequent variants (MAF < 1.5). All variants were manually assessed before inclusion in the final report.

Trio-WES was performed for patient 2 and his parents. Exome enrichment, including library preparation, was performed with the SureSelect Human All Exon Kit V6 using the Bravo Automated Liquid Handling Platform according to the manufacturer's specifications (Agilent Technologies, Santa Clara, CA). Next-generation sequencing on the Illumina NovaSeq6000 was conducted in 2 × 100 bp paired-end mode (Illumina, San Diego, CA). Overall coverage of 117× for the child was achieved with >99% of the reads reaching an individual coverage of >20×. Reads were aligned to the human genome build (GRCh37/hg19). Variants were filtered by minor allele frequency in the Genome Aggregation Database (9) by mode of inheritance, and by predicted functional impact using the VarFish platform (10). The pathogenicity of identified variants was evaluated using MutationTaster (http://www.mutationtaster.org), Polyphen-2 (11), SIFT (12), and by CADD score. The splicing effect was assessed using varSEAK Online (https://varseak.bio/) and the scores of SpliceAI and MMsplice. Furthermore, a comparison with the mutation databases ClinVar (http://www.ncbi.nlm.nih.gov/clinvar/) and the HGMD (HGMD Professional 2021.1) was performed. Candidate variants were classified according to the ACMG guidelines (13).

## 3. Discussion

Here, we present the clinical and molecular data of five patients from three unrelated families with three different pathogenic *KPTN* variants (Table 1). So far, severe developmental delay and primary, or more frequently secondary, macrocephaly (with a range up to +5.4 SD) have been reported as the consistent features in 14/14 published affected individuals with biallelic pathogenic variants in *KPTN* (5). Our data with the lack of macrocephaly in one patient indicate that macrocephaly does not occur in all patients with this syndrome.

Epilepsy has been reported to occur in one-third of affected individuals, varying with respect to seizure types and severity (5). GTCSs are the most frequently described seizure type, starting between 3 months and 7 years of age (5). Detailed data of ASM of these reported individuals have not yet been published. Here, we report epilepsy in two of five patients. Patients 1 and 2 of this study have developmental and epileptic encephalopathies (DEEs) with an early diagnosis of a developmental delay and seizure onset at the age of 5 years and 13 months, respectively. The other three patients had not developed epilepsy at the age of 6, 9, and 13 years, respectively. They hold a developmental delay phenotype. So far, only one patient with MRT41 carrying the compound heterozygous variants p.(Met241_Gln246dup) and c.394+1G>A was reported with intractable epilepsy and had died at the age of 9 years due to SE (3).

Structural brain anomalies may be part of the phenotypic spectrum. Various brain anomalies such as ventriculomegaly and non-specific supratentorial leukoencephalopathy have been documented in half of the reported affected individuals, but none has been reported in several individuals frequently. Here, we report novel MRI findings of optic nerve tortuosity and signs of intracranial hypertension in patients 1 and 2 (flattened pituitary gland in patient 1 and mild enlargement of the inner cerebrospinal fluid spaces in patient 2). Intracranial hypertension, in patient 2 confirmed by lumbar puncture and to a mild degree in patient 1, has not previously been reported in association with pathogenic biallelic variants of *KPTN*.

More than half of the reported patients developed behavioral abnormalities such as repetitive speech, stereotypic movements, hyperactivity, and autistic features (1, 5). Patients 1, 2, and 3 of this study presented similar behavioral problems. Frontal bossing and prominent chin are the most frequently described facial anomalies in this condition, also present in patients 1 and 2 (frontal bossing) and patient 3 (prominent chin) reported here (5).

The heterozygous frameshift variant p.(Ser200Ilefs^*^55) found in patient 1 has been reported in two affected families of different populations (4, 5). The second variant, p.(Met241_Gln246dup), detected in patient 1 has been described in a compound heterozygous state in two other affected families and is shown to have a profound effect on kaptin function (1, 3). This in-frame duplication in compound heterozygous status is associated with a wide range of severity from status epilepticus to absence seizures (1, 3). Comparing the data on all published patients, there is no association between a specific mutation in *KPTN* and the severity of epilepsy. The homozygous *KPTN* variant c.599+1G>A, affecting a splice site, occurred in the unrelated families 2 and 3 of this study but has not been reported previously in the literature.

In conclusion, our data expand the phenotypic and mutational spectra of this rare syndrome. We highlight the severe epilepsy phenotype with DEE in two of the five reported children. Moreover, we challenge the presence of macrocephaly in all individuals with MRT41 and introduce as a putative feature an increased opening pressure. Mutated components of the protein complex KICSTOR such as KPTN are known to cause hyperactive mTOR signaling (5). The disturbed mTOR signaling opens new therapeutic options for these patients through the application of known mTOR inhibitors.

## Data availability statement

The original contributions presented in the study are publicly available. This data can be found here: [https://www.ncbi.nlm.nih.gov/clinvar/; accession numbers: SCV001334672.11, SCV001747927.6 and SCV002574953.1].

## Ethics statement

Written informed consent was obtained from the minor(s)' legal guardian/next of kin for the publication of any potentially identifiable images or data included in this article.

## Author contributions

SH wrote the manuscript and collected the clinical data. MD, FB, and SB performed the molecular and genetic analysis. NE, CPo, and CPr delivered further clinical data, especially the EEG analysis and EEG results. AK supervised the manuscript. All authors contributed to the article and approved the submitted version.
