# Supplementary material for: Case report: KPTN gene-related syndrome associated with a spectrum of neurodevelopmental anomalies including severe epilepsy
Source: Front Neurol. 2023 Jan 10;13:1113811. doi: 10.3389/fneur.2022.1113811 (PMC9871926; doi:10.3389/fneur.2022.1113811)
Supplement: Supplementary file 1 [file Data_Sheet_1.PDF]

Supplementary Material

Case Report: KPTN gene-related syndrome associated with a spectrum of neurodevelopmental anomalies including severe epilepsy

Svea Horn*, Magdalena Danyel, Nina Erdmann, Felix Boschann, Cecilia Gunnarsson, Saskia Biskup, Jerome Juengling, Cornelia Potratz, Christine Prager, Angela M. Kaindl

*** Correspondence:** Prof. Dr. Angela M. Kaindl, Pediatric Neurology, Charité – Universitätsmedizin Berlin, Campus Virchow-Klinikum, Augustenburger Platz 1, 13353 Berlin. Tel / Fax: +49 30 450 566301 /7566301. Email: angela.kaindl@charite.de.

1 Supplementary Data

Clinical report

Patient 1

According to ACMG classifications both variants (c.597_598dupTA; p.Ser200Ile*fs**55 and c.714_731dup; p.Met241_Gln246dup) were classified as pathogenic (first: PVS1, PS4_sup, PM2_sup, PP4 ; second: PS4, PM3, PM4, PP4).

Patient 2

According to ACMG framework we classified the variant (c.599+1G>A; p.?) as pathogenic (PVS1, PS4_mod, PM2_sup, PM3_sup, PP4).
